# Predictors of quality of life in a longitudinal study of users with severe mental disorders

**DOI:** 10.1186/1477-7525-11-92

**Published:** 2013-06-08

**Authors:** Marie-Josée Fleury, Guy Grenier, Jean-Marie Bamvita, Jacques Tremblay, Norbert Schmitz, Jean Caron

**Affiliations:** 1 Department of Psychiatry, McGill University, Douglas Mental Health University Institute, 6875 LaSalle Blvd., Montreal, Quebec H4H 1R3, Canada; 2 Douglas Hospital Research Centre, 6875 LaSalle Blvd., Montreal, Quebec H4H 1R3, Canada

**Keywords:** Subjective quality of life, Needs, Social support, Severe mental disorders

## Abstract

**Background:**

Since the end of the 20th century, quality of life has become a key outcome indicator in planning and evaluation of health services. From a sample of 297 users with severe mental disorders from Montreal (Canada), this study aimed to identify the key predictors of subjective quality of life (SQOL).

**Methods:**

Users were recruited and interviewed from December 2008 to September 2010 and re-interviewed approximately 18 months later. A comprehensive framework including socio-demographic data, clinical, needs and functionality variables, negative life events, social support and healthcare service use, and appreciation data were considered as predictors. Clinical records and eight standardized instruments were used.

**Results:**

Lower severity of needs, schizophrenia, better social integration, better reassurance of worth, fewer drug abuse problems, and living in supervised housing are predictors of SQOL. With regard to needs, absence or lower severity of needs in the areas of company, daytime activities, social exclusion, safety to self, and benefits are linked to SQOL.

**Conclusion:**

Reducing the severity of needs is especially beneficial to ensure a higher SQOL for users with severe mental disorders. To improve SQOL, priority must be given to programs and interventions that promote the development of a stimulating and supportive social network, and maintain a plurality of residential services matching the functional abilities of users.

## Background

Since the end of the 20th century, quality of life (QOL) has become a key outcome indicator in planning and evaluation of health services in general [1-4], and primarily for chronic disorders, such as severe mental disorders, which require treatment over a long period [5]. The first mental health studies of QOL [6,7] occurred at a time when it became necessary to expand community services for users discharged from psychiatric hospitals.

QOL has subjective and objective components. The subjective component refers to “well-being,” “happiness” or “life satisfaction,” whereas the objective component refers to aspects of environment and social functioning [2]. Several studies had shown discrepancies between subjective (SQOL) and objective QOL [6-11]. Most studies focus on SQOL, which is influenced by multiple factors [12]. SQOL is closely linked to the recovery paradigm, which oriented most of the mental healthcare reforms in industrial countries [13,14]. For the health system, recovery is a goal to reach, and it is defined as “a way of living a satisfying, hopeful, and contributing life even with limitations caused by the illness. It involves the development of new meaning and purpose in one’s life as one grows beyond the catastrophic effects of mental illness” [15].

Cross-sectional and comparative studies have identified several determinants of SQOL among users with severe mental disorders. Those studies found that SQOL is strongly associated with clinical variables [3,4,16-20] and perceived needs [21-30]. Mood disorders are the most influential determinant of lower SQOL [16]. Regarding users with schizophrenia, a lower SQOL was associated with the presence of a second diagnosis such as major depression [3,4,17-20,31,32], and anxiety [4,18,19,33]. Users with less severe psychiatric symptoms [1,34] and better cognitive performance [20] presented a higher SQOL. Meanwhile, past negative life events [17] produced a lower SQOL [32], especially among crime victims [3] and users with repeated suicide attempts.

Most studies have found a strong correlation between unmet needs (serious problems) and poorer SQOL [24,25,28-30]. A higher number of needs (met or unmet) is associated with a poorer SQOL [24,35]. Lower SQOL is most often associated with unmet social needs (intimate relationship, sexual expression, and especially company). Unmet basic and functioning needs are also associated with lower SQOL [25]. Other studies found an association with lower SQOL and unmet needs in daytime activities [24,26], childcare [24,30], psychological distress [26], accommodation, psychotic symptoms and benefits areas [30].

Relationships between SQOL and social support have also been the subject of several studies [36-38]. SQOL is significantly associated with the availability and adequacy of various social relationships [36], and social networks [10,37]. Among various components of social support, attachment and reassurance of worth have a significant link with SQOL [37]. Stigma and self-stigma are social variables negatively associated with SQOL [39,40].

Socio-demographic variables are less strongly correlated with SQOL than clinical and need variables or social support [2,19,41]. However, SQOL is usually higher among users having a higher income [37] or a job [42,43]. Women show a higher SQOL than men [44]; the same is true for older versus younger users [45]. Quality of housing still has a crucial impact on SQOL [46-48]. According to the literature, autonomous accommodations are the best type of housing for users with severe mental disorders [47]. Supervised housing, however, is satisfactory for a substantial proportion of users [21,49,50].

The association between SQOL and use of mental health services is not as well covered in the literature. Antipsychotic medication compliance presents two opposing effects on SQOL. By reducing symptoms, medication increases SQOL; however, side effects are associated with a lower SQOL [22]. SQOL is also linked with greater satisfaction with mental healthcare [23].

Longitudinal analysis offers a stronger method than cross-sectional analyses for examining the relationship between SQOL and related variables [33]. Several longitudinal studies have investigated predictors of SQOL in users with severe mental disorders [4,7,11,12,19,24-33,35,38],[41,51-58], primarily schizophrenia [11,19,24,28,31-33,35,38,41],[52,54-58]. Studies concerning perceived needs have found that quality of life improves when the number of serious needs decreases [24-26,29,30,38]. Other studies have noted that SQOL rises when symptom severity is reduced and functional ability is improved [11,12,24,28,33,35,41,54],[56,58]. A reduction in stress-related factors [56] or in substance abuse [12] also improves SQOL. A stronger social network is also a significant predictor of a higher SQOL in these longitudinal studies [12,28,38]. Finally, some studies reported an improvement of SQOL among users with severe mental disorders after their discharge from hospital [7,10]. Other predictors of change in SQOL longitudinal studies are service use [12] and satisfaction with services [4,58].

While some studies have longitudinally assessed the SQOL of users with severe mental disorders, most of them have gaps. First, in most longitudinal studies, the sample consists mainly or exclusively of users suffering from schizophrenia. Few studies have compared SQOL among a heterogeneous cohort of users with severe mental disorders living in the community. Second, studies investigating associations between perceived needs and SQOL have generally not included other factors in their analysis and, conversely, perceived needs were usually not considered in studies assessing clinical, socio-demographic, social support or service use as predictors of SQOL. Third, some variables (e.g. socio-demographic variables such as education or employment, negative life events) that could change SQOL are analyzed only in cross-sectional studies or not considered at all.

Based on a longitudinal study and a comprehensive conceptual framework, including variables less commonly studied in longitudinal research, this study aimed to determine 1) predictors of SQOL of a heterogeneous cohort of users with severe mental disorders over an 18-month period, and 2) areas of need that are the main predictors of the SQOL among this clientele living in the community. Based on the literature on the relationship between needs and SQOL, we hypothesized 1) that the severity of needs would be the strongest SQOL predictor for these users; and 2) that out of the needs categories, social needs would be the best predictor of higher or lower SQOL.

## Methods

### Study design and users selection criteria

This cohort study was conducted at a mental health university institute (MHUI – offering specialized services) and two health and social service centers (HSSC – offering primary mental healthcare), located in the southwestern area of Montreal, Canada. This urban area serves a population of 258,000. The study involved a two-time measurement. Users with severe mental disorders were first randomly recruited at baseline, and then re-interviewed approximately 18 months later. They were aged from 18 to 65 years, diagnosed with a severe mental disorder according to the DSM-IV [59] diagnosis criteria 147 (schizophrenia and other psychotic disorders) or 161 (mood disorders). They resided in the zone covered by the study and received follow-up services at the MHUI or one of the two HSSC. They agreed to let the research team access their medical records and contact their case manager for the purpose of filling out a questionnaire on their functional ability in the community. Users with severe mental retardation, those following mandatory psychiatric treatment as determined by a judiciary board, and those with a history of hospitalization or emergency room visit in the three months prior to the initial interview were deemed unable to complete the questionnaire and were thus excluded from the study.

### Data collection

User recruitment involved various strategies (posters for participant self-referral, recruitment at out-patient clinics, and information sessions or flyers to explain the project). Data was collected from December 2008 to September 2010 (baseline, T0), and from January 2011 to November 2011 (T1). Each user participated in two 90-minute interviews, conducted at a week’s interval by a team of specially trained clinical professionals, and monitored by a research coordinator. Users’ medical records were also reviewed. Interviews were based on eight questionnaires, seven of which were administered to users, and one (the Multnomah Community Ability Scale- MCAS) was completed by users’ case managers (Table 1). Questionnaires were administered in English or French, according to the user’s spoken language. With the exception of self-referral, users were contacted by their case managers who referred potential participants to the research team when appropriate. Each participant provided informed consent. The study protocol was approved by the MHUI and the two HSSC ethics review boards.

**Table 1:** Standardized instruments used

**Name**	**Description**	**Variables**
Satisfaction with Life Domains Scale (SLDS) [6]	Assesses satisfaction in 20 life domains; seven-point Likert scale, questions; Cronbach alpha: 0.92 [60]	Subjective quality of life
Montreal Assessment of Needs Questionnaire (MANQ) [61]	Derived from the Camberwell Assessment of Need (CAN) Assesses user needs in 26 needs areas; 11-point analog scales (0–10); Cronbach Alpha: 0.70 to 0.73	Age
		Gender
		Civil status
		Education
		Employment
		Type of housing (autonomous/supervised)
		Nationality (Canadian/Others)
		Spoken language (French/Others)
		Importance attributed to spirituality
		Number of needs areas (26 in five categories: basic; health; functioning; social, services)
		Severity of need
		Amount of help received from relatives
		Amount of help received from services
		Adequacy of help received
Social Provision Scale (SPS) [62]	Measures availability of social support in six dimensions; Cronbach Alpha: 0.92 [62]	Attachment, reassurance of worth, social integration, reliable alliance, guidance and nurturance
Multnomah CommunityAbility Scale (MCAS) [63]	Assesses user’s functional ability in the community, e.g. obstacles to functioning, social competences; Cronbach Alpha: 0.87 [63]	Functional ability in the community
Alcohol Use Disorders Identification Test (AUDIT) [64]	Measures alcohol consumption level and consequences; 10 items; yes/no questions; Cronbach Alpha: 0.88 [65]	Alcohol abuse
Drug Abuse Screening Test (DAST-20) [66]	Measures user’s drug use and consequences; 20 items; yes/no questions; Cronbach Alpha: 0.74 [66]	Drug Abuse
Alberta Continuity of Services Scale for Mental Health (ACSS-MH) [67]	Measures service continuity, e.g. system access and team function; Cronbach Alpha: 0.78 to 0.92	Service continuity
Service Utilization Questionnaire (SUQ) [68]	Derived from the Canadian Health Survey Questionnaire (CCHS) Evaluates types and frequency of professionals and services used	Visit to any healthcare professional or services
Clinical records	Diagnoses	Schizophrenia
		Mood disorders
		Anxiety disorders
		Personality disorders
		Delusion and other psychotic disorders
		Schizophrenia spectrum disorders
		Number of mental disorders
		History of prior violence
		History of prior judiciary problems Being on medication

### Measurement instruments

SQOL was measured with the modified version of the Satisfaction with Life Domains Scale (SLDS), initially developed by Baker and Intagliata [6], and translated in French and validated (Cronbach Alpha = 0.92) by Caron et al. [60]. The SLSD assesses satisfaction regarding 20 life domains. Users had to choose among seven stylized faces ranging from 1 to 7, from the happiest face (score 7) to the saddest one (score 1) that corresponded to their emotional state [36,69,70]. All item scores were added up to arrive at a total score. The psychometric properties of the SLDS are good, which makes it an effective instrument to measure SQOL. The SLDS is among the scales that were included in their entirety in the Wisconsin Quality of Life index, a multidimensional instrument containing 113 items [71,72].

The second major questionnaire used was the Montreal Assessment of Needs Questionnaire (MANQ), derived from the Camberwell Assessment of Need (CAN). The CAN is one of the most widely used instruments for needs assessment (Cronbach Alpha = 0.64 for total needs) [31]. It assesses user needs in 22 areas, divided into five categories: basic, health, functioning, social and service needs. The CAN evaluates needs by taking into account their number and their severity. For the MANQ (Cronbach Alpha = 0.70 to 0.73), four areas were added: stress adaptation (included as a health need), social exclusion (social need), involvement in treatment decisions (service need), and job integration (basic need) for a total of 26 areas (Table 1). Unlike the CAN, which assesses the interviews and measures users’ needs with ordinal scales (0 to 2; or yes/no), the MANQ uses 11-point analog scales, ranging from 0 (no need) to 10 (severe need). Users answer questions with the help of the interviewers. These changes reflect the new recovery paradigm that is at the heart of current mental health reforms [13,14] and enhance data variability. The MANQ also includes items related to socio-demographic and socio-economic user profiles. The validation of the MANQ was the subject of a previous publication [61].

The six other standardized instruments used for this study both at T0 and TI are described in detail in Table 1: the Social Provisions Scale (SPS), the Multnomah Community Ability Scale (MCAS), the Alcohol Use Disorders Identification Test (AUDIT), the Drug Abuse Screening Test-20 (DAST-20), the Alberta Continuity of Services Scale for Mental Health (ACSS-MH), and the Service Utilization Questionnaire (SUQ).

### Analysis and conceptual framework

To reduce errors in building data files, questionnaire data were collected scanning with TELFORM® software, and automatically loaded in SPSS files. A research assistant validated each file entry. Data was thoroughly cleaned before going through analyses. Verification of normality assumptions for every continuous variable revealed that no variable transformation was necessary.

**Figure 1:**
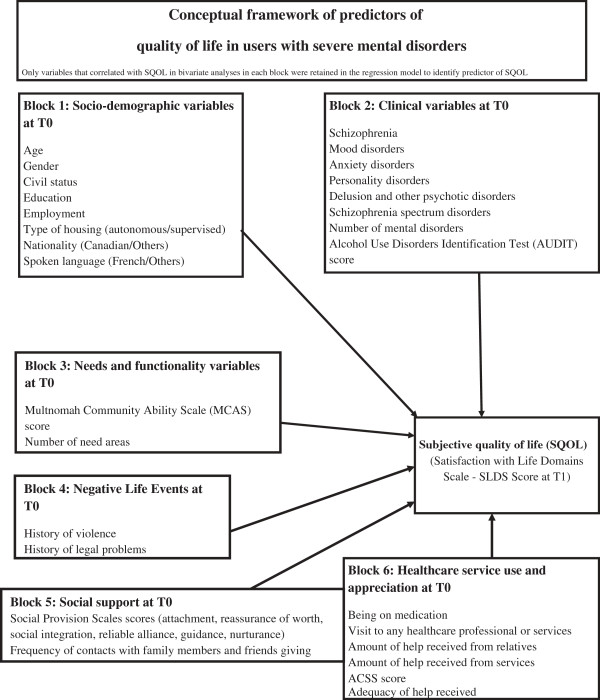
Conceptual framework of predictors of quality of life in users with severe mental disorders.

Analyses followed the conceptual framework displayed on Figure 1, based on previous research and especially on Caron’s SQOL framework [73]. The dependent variable was the SQOL, measured with the SLDS instrument at T1. Predictors (N = 32), collected at T0, were organized in six categories: socio-demographic variables, clinical variables, needs and functionality variables, negative life events variables, social support variables, and healthcare service use and appreciation variables. The chosen predictors take into consideration all the aspects that, according to previous studies, could influence a change in SQOL among users with severe mental disorders. As regards scores from the Social Provision Scale, sub-scale scores allowed subtler analyses based on the categorization by Cutrona and Russell [62] in attachment, social integration, reassurance of worth, reliable alliance, guidance, and opportunity for nurturance.

Univariate analyses comprised frequency distribution for categorical variables and mean values for continuous variables to describe participants in the sample. A hierarchical linear regression model was used for an Alpha value set at 0.05. The hierarchical model permitted to test the conceptual framework. The six blocks of predictors displayed in Figure 1 were entered successively, as indicated by their number. The blocks that were most reliable and less subject to interpretation were entered first (e.g. socio-demographic variables, followed by clinical variables). Only variables that had correlated with SQOL in bivariate analyses in each block were retained in the regression model. Goodness-of-fit and total variance explained were generated for each block as well as the entire model. In order to avoid multi-colinearity between explanatory variables, their relationships were checked using two tests: Collinearity diagnostic and Tolerance. Tolerance is the opposite of the variance inflator factor (VIF). A VIF close to the 10.0 is a reflection of collinearity between variables, as is a tolerance close to 0.1. Lastly, further analysis consisted in assessing predictors of SQOL by taking into account only needs variables. A hierarchical linear regression model was also used for this purpose.

## Results

### User sample

Overall, 437 users were recruited at baseline (T0), with 352 (80.5%) consenting, all of whom were contacted for a second series of interviews 18 months later (T1). The characteristics of the users recruited at T0 were described in a previous study [74-76].

A total of 297 users (84.4%) agreed to participate at T1, whereas 16 (4.5%) users refused to do so, seven (2.0%) were excluded due to incapacity, six (1.7%) were deceased, and 26 (7.4%) had moved outside of the study area or could no longer be found. Comparison analyses between the 55 users who did not take part in the follow-up survey and the remaining 297 participants yielded no significant differences in terms of age distribution (P = 0.409), gender distribution (*X*^2^ = 0.325, P = 0.58), or type of housing (autonomous versus supervised: *X*^2^ = 0.406; P = 0.524). Participants who took part in both the baseline (T0) and follow-up (T1) surveys were also compared to those who responded only to the baseline assessment with respect to the most frequent mental health disorders, and no significant differences were found (schizophrenia: *X*^2^ = 0.043, P = 0.835; schizophrenia spectrum disorders: *X*^2^ = 0.015, P = 0.901; mood disorders: *X*^2^ = 2.170, P = 0.141; delusion disorders: *X*^2^ = 0.259, P = 0.611; anxiety disorders: *X*^2^ = 0.547; P = 0.460).

In total, 153 males (51.5%) and 144 females (48.5%) participated in the study, with a mean age of 48 years (SD: 10.4), as shown in Table 2. A majority of users (64.0%) were French-speaking and single (75.4%). Two-thirds had completed primary or secondary school (62.3%). Most users (60.5%) lived in autonomous housing. The most prevalent severe mental health disorders were mood disorders (41.8%), followed by schizophrenia (38.0%), schizophrenia spectrum disorders (12.8%) and delusion and other psychotic disorders (9.4%). Most participants had also a second diagnosis of mental disorder, the most common being a personality disorder (27.3%). The majority of users suffering from schizophrenia (N = 73 or 61.9%) lived in supervised housing, and most users having another severe mental disorder lived in autonomous housing (N = 136 or 74.7%).

**Table 2:** Socio-demographic, socio-economic and clinical variables (N = 297)

**Categories**	**Sub-categories**	**Variables**	** n**	** %**
**Sociodemographic variables**	**Age [Mean (SD)]**		48.5	10.4
	**Gender**	Men	153	51.5
		Women	144	48.5
	**Spoken language**	French	190	64.0
		English	61	20.5
		French/English	7	2.4
		Others	37	12.5
	**Civil status**	Single/Never married	224	75.4
		In a relationship/Married/Remarried	33	11.1
		Separated/Divorced/Widow	40	13.5
**Socio-economic variables**	**Type of housing**	Autonomous housing	179	60.3
		Supervised housing	116	39.1
	**Education**	Primary/Secondary school	185	62.3
		College/University	110	37.0
**Clinical variables**	**Severe mental health disorders**	Schizophrenia	113	38.0
		Mood disorders	124	41.8
		Schizophrenia spectrum disorders	38	12.8
		Delusional and other psychotic disorders	28	9.4
	**Second diagnosis**	Anxiety disorders	36	12.1
		Dependences and substance abuse		
		Drug	16	5.4
		Alcohol	13	4.4
		Multiple	27	9.1
		Personality disorders	81	27.3
		Moderate or mild mental retardation	37	12.5

Table 3 shows the number and severity of user needs. The five most reported needs were psychological distress (68.7%), stress adaptation (61.3%), physical health (56.6%), psychotic symptoms (48.5%), and money (47.1%). In regard to the severity of needs, the highest means were related to job integration (mean: 7.5), childcare (7.5), drugs (7.4), sexual expression (7.4) and intimate relationships (7.3).

**Table 3:** Number and severity of needs of participants according to the MANQ (N = 297)

**Categories**	**Areas**	**N**	**%**	Severity Mean*	**SD**
Basic	Job integration	101	34.0	7.50	2.504
	Accommodation	101	34.0	5.96	3.062
	Daytime activities	98	33.0	5.52	2.729
	Food	96	32.3	5.24	2.396
Health	Psychological distress	204	68.7	6.67	2.732
	Stress adaptation	182	61.3	6.30	2.599
	Physical health	168	56.6	5.70	2.851
	Psychotic symptoms	144	48.5	5.54	2.715
	Drugs	99	33.3	7.44	3.035
	Safety to self	58	19.5	6.00	2.853
	Alcohol	31	10.4	5.10	2.371
	Safety to others	22	7.4	5.77	3.206
Functioning	Money	140	47.1	6.84	2.904
	Looking after the home	99	33.3	5.69	2.448
	Self-care	49	16.5	5.55	2.829
	Basic education	42	24.1	5.79	3.000
	Childcare	21	7.1	7.43	2.993
Social	Sexual expression	122	41.1	7.42	2.787
	Company	101	34.0	4.06	3.736
	Intimate relationships	97	32.7	7.30	2.84
	Social exclusion	95	32.0	6.16	2.841
Services	Transportation	65	21.9	7.12	3.059
	Information about illness and treatment	65	21.0	5.94	2.800
	Involvement in treatment decisions	58	19.5	6.12	2.986
	Benefits	30	10.1	6.87	3.126
	Telephone	8	2.7	6.38	2.615

* Severity range from 0 to 10 (10 was the highest).

### SQOL predictors

Table 4 shows the hierarchical linear regression model. The category related to needs and functionality variables is the best predictor of QOL. Two variables, severity of needs and MCAS scores, together accounted for 11.8% of QOL and had a negative impact on QOL. However, the MCAS beta scores were not significant in final analyses. The socio-demographic variables were the second best predictor of QOL, accounting for nearly 8% of the variance; autonomous housing was retained, and it was found to have a negative impact on QOL. Social support variables accounted for 7.9% of the variance; perception of availability of social integration and reassurance of worth supports had a positive link with QOL. Finally, two clinical variables explained 4.9% of the variance: a diagnosis of schizophrenia had a positive link to QOL while DAST-20 scores were negatively related. No variables were retained from the fourth (negative life events) and sixth (healthcare service use and appreciation) blocks. For the entire model, the total variance explained was 37%, with an acceptable goodness-of-fit (P < 0.001). No sign of multi-colinearity was detected (tolerance coefficients were over 0.7, and VIF coefficients were under 2.0).

**Table 4:** Predictors of SQOL in users with mental disorders: Hierarchical linear regression analysis

	**Sociodemographic variables**		**Clinical variables**		**Needs and functionaliy variables**		**Social support**						
**Predictors from baseline**	**β**	**P**	**β**	**P**	**β**	**P**	**β**	**t**	**P**	**95% CI**		**95% CI Colinearity statistics**	
										LL	UL	Tolerance	VIF
Type of housing (autonomous)	-.282	.000	-.199	.001	-.151	.008	-.160	−2.937	.004	−10.713	−2.116	.787	1.271
Schizophrenia			.181	.002	.155	.006	.166	3.142	.002	2.497	10.873	.837	1.195
DAST-20 score			-.147	.008	-.074	.157	-.101	−2.020	.044	−1.395	-.018	.934	1.071
Severity of needs					-.330	.000	-.272	−5.176	.000	-.226	-.101	.847	1.180
MCAS score					.111	.036	.079	1.566	.118	-.041	.361	.920	1.086
Social integration							.196	3.472	.001	.942	3.410	.732	1.366
Reassurance of worth							.136	2.397	.017	.255	2.597	.721	1.386
Total variance explained (R2)	8.0%		12.9%		24.7%		32.6%						
Goodness-of-fit													
F	25.518		14.523		19.133		19.940						
P	<0.001		<0.001		<0.001		<0.001						

Table 5 shows the areas of need identified as SQOL predictors. Health needs account for 9.7% of the variance of the model. It includes safety to self, stress adaptation and drugs. However, only safety to self retained a significant beta in final analyses and was negatively related to SQOL. However drugs showed a tendency to be positively related to SQOL. Service needs accounted for 8.2% of the variance, but only benefits showed a tendency to be negatively related to SQOL in the final analyses. Social needs explained 5.4% of the variance; need for company and social exclusion had a negative relation to SQOL. Among the basic needs that explained 5.2% of the variance, daytime activities was the only negative predictor of SQOL. Finally, among basic needs (1.5%), only self-care showed a negative relationship in the initial analyses, but its beta was no longer significant in the full model. The total variance explained was 30%, with an acceptable goodness-of-fit (P < 0.001). No sign of multi-colinearity was detected (tolerance coefficients were over 0.7, and VIF coefficients were under 2.0).

**Table 5:** Severity of needs predicting SQOL in users with mental disorders: Hierarchical linear regression analysis

	**Services**		**Functioning**		**Health**		**Basic**		**Social needs**						
	**b**	**P**	**b**	**P**	**b**	**P**	**b**	**P**	**b**	**t**	**P**	**95cpc CI**		**Colinearity statistics**	
												LL	UL	Tolerance	VIF
Benefit	-.185	.001	-.191	.001	-.146	.009	-.126	.020	-.101	−1.936	.054	−1.740	.014	.895	1.117
Involvement in treatment decisions	-.185	.001	-.177	.002	-.112	.044	-.082	.131	-.055	−1.051	.294	−1.127	.342	.878	1.139
Self-care			-.122	.029	-.097	.070	-.064	.222	-.020	-.382	.703	−1.014	.685	.902	1.109
Safety to self					-.205	.000	-.190	.000	-.155	−2.940	.004	−1.878	-.372	.880	1.137
Stress adaptation					-.173	.002	-.104	.066	-.030	-.531	.596	-.753	433	.757	1.320
Drugs					.126	.020	.102	.052	.091	1.803	.072	-.042	.952	.950	1.052
Daytime activities							-.251	.000	-.183	−3.171	.002	−1.908	-.447	.736	1.358
Company									-.225	−3.730	.000	−1.843	-.570	.673	1.486
Social exclusion									-.113	−2.057	.041	−1.312	-.029	.811	1.233
Total variance explained (R2)	8.2%		9.7%		19.4%		24.6%		30.0%						
Goodness-of-fit															
F	13.107		10.467		11.664		13.503		13.646						
P	<0.001		<0.001		<0.001		<0.001		<0.001						

*Variables entered into blocks*: **Block 1 or Services**: Information about illness and treatment, telephone, transport, benefits, involvement in treatment decisions; **Block 2 or Functioning**: Looking after the home, self-care, basic education, money, childcare; **Block 3 or Health**: Psychotic symptoms, physical health, drugs, alcohol, safety to self, safety to others, psychological distress, stress adaptation; **Block 4 or Basic needs**: Accommodation, food, daytime activities, job integration; **Block 5 or Social needs**: Company, intimate relationships, sexual expression, social exclusion.

## Discussion

This article aimed to identify 1) the predictors of SQOL of a heterogeneous cohort of users with severe mental disorders over an 18-month period, and 2) the areas of need that are the main predictors of the SQOL. We hypothesized that the severity of needs would be the strongest SQOL predictor for these users, and out of the needs categories, that social needs would be the best predictors of SQOL.

Overall, our conceptual framework predicted SQOL at an acceptable level (33% of the variance explained) — comparable to or higher than most SQOL studies — especially considering the diversity of the sample, which reflected the full spectrum of users with severe mental disorders living in the community [3,18,77]. In accordance with our first hypothesis, the strongest predictor of SQOL was found to be the severity of needs: the less severe the needs, the higher the SQOL. This result compares with previous studies, which found that unmet needs (serious problems) [24,78-80] had a highly negative impact on SQOL. Findings using the CAN as the needs instrument made a distinction between serious problems, moderate problems and absence of needs [24,47,81,82]. In our study, we changed this ordinal scale to an 11-point analog scale, ranging from 0 to 10. Our results confirm that SQOL is influenced by serious problems, but also by moderate problems in some needs areas. Furthermore, in contrast to previous studies [26,28], we did not find a significant association between SQOL and the number of needs. This could indicate that some needs areas (e.g. company) may have a greater impact on SQOL than others.

SQOL is associated with two areas of social support: reassurance of worth and social integration. Reassurance of worth indicates acknowledgement of competence and skills by others [62]. According to the literature, reassurance of worth is an exceptionally strong predictor of SQOL among users with severe mental disorders, as it is among poorer groups and the general population [37,83]. Social integration indicates that the individual shares common interests and activities with a network of friends [66,84], and is less likely to experience loneliness or social isolation [84].

Two clinical variables were identified as predictors of SQOL: schizophrenia and lower drug use and consequences according to the DAST-20 scores. The link between a schizophrenia diagnosis and higher SQOL was unexpected. It could be explained by the fact that users with schizophrenia constituted a minority of our sample and that most of them had no other mental disorder. Most studies concerning SQOL included mainly or exclusively users with schizophrenia, with some of them also presenting a second diagnosis, such as a mood disorder. Furthermore, a previous study by Caron et al. [37] found that users with schizophrenia reported a comparable SQOL to the general population. Their level of satisfaction resulted from community mental health programs, access to supervised apartments, higher welfare benefits compared to non-disabled recipients, and ability to rely on professionals for advice and emotional support. Users with schizophrenia may also be more satisfied with their SQOL because they have fewer personal projects and less desire for change than users suffering from other severe mental disorders [45]. SQOL is thus independent of the standard of living (Objective QOL) among users with schizophrenia [85]. Users with chronic schizophrenia can be satisfied with their SQOL despite living conditions that would be considered unpleasant to clinicians or the general population [86]. Additionally, according to Ruggeri et al. [18], users with schizophrenia tend to overestimate their level of functioning. The association between reduced drug use and consequences according to the DAST-20 score and better SQOL makes sense. Dual diagnoses usually result in poorer outcomes [87]. Substance abuse increases violence and medication non-compliance among users with severe mental disorders, and exacerbates the actual symptoms of schizophrenia [27]. Users with a dual diagnosis have a poorer SQOL than those who have only a single diagnosis of either severe mental disorder or substance abuse [27,88].

A single socio-demographic variable had any kind of connection with SQOL: users living in supervised housing reported a better SQOL. This result seems in opposition with literature that has reported a link between SQOL and autonomous housing [47]. Some previous studies, however, have reported that supervised housing positively influences SQOL [21,49]. Indeed, according to the residential continuum model, users are matched with the type of residential services corresponding to their service needs and psychiatric impairment, and are transferred progressively into more autonomous housing situations if and when their condition improves [48,50]. Some previously hospitalized users may be satisfied with living in supervised housing [21]. Furthermore, according to Priebe [86], comparing one’s living situation with original aspirations and expectations, matching up one’s own position with that of others, and adaptation over time are the three main processes that influence one’s perceived SQOL. It may be that users living in supervised housing express few worries or little desire for change when comparing their housing situation to that of persons living in autonomous housing because some of their previous goals have become less relevant over time [21,45]. It could be the case of users with schizophrenia versus those affected by mood disorders or other severe mental disorders. In our sample, most users with schizophrenia lived in supervised housing. Lastly, when compared to users living in autonomous housing, users living in supervised housing are less likely to experience social loneliness.

In opposition to our second hypothesis according to which social needs would be the stronger SQOL predictors, it was health needs that contributed the most to the model. However, the only health need area associated with SQOL is safety to self. The link between SQOL and a lack of needs or rare serious problems in the area of safety to self is logical. Safety to self is one of the most common needs among users with a history of suicide attempts [89].

Table 4 confirms the importance of social needs areas as SQOL predictors. According to Lasalvia et al. [24], a reduction of unmet social needs predicts an increase in the SQOL. Of the four areas of needs that have a negative impact on SQOL, two are in the social category: company and social exclusion. The absence of needs or few serious problems in those two areas are signs of genuine social integration, which is essential to a higher SQOL [66]. As noted in previous studies, company is the area of need most strongly associated with a poorer SQOL [24,26,82].

Daytime activities are a basic need, but the absence of needs or few serious problems in that area are also indicative of healthy social integration. Daytime activities help to build social networks and create friendships. According to previous studies with the CAN using factor analysis, daytime activities and company are often included in the same need factor [90,91]. Benefits are a service need but are also indirectly associated with reassurance of worth and social integration. Reassurance of worth is lower among users who do not have a meaningful economic role [37]; such is the case for those expressing a need in the area of benefits. Social loneliness and stigmatization often occur among users who need benefits. Like daytime activities, employment helps to develop a social network [92].

Finally, it is interesting to note that needs with higher average severity (job integration, childcare, etc.) do not significantly alter SQOL. Conversely, the need for company is the one having the lowest average severity. This indicates that the presence of a moderate problem in this area is sufficient to affect SQOL significantly.

## Limitations

Our study includes certain notable limitations. First, time from T0 to T1 was relatively short (18 months). Secondly, as our sample represented a heterogeneous group of users with severe mental disorders (which is also a strength), the results may not be applicable to a sample constituted exclusively of users with schizophrenia or other specific diagnosis. Third, the study did not take into account the duration of the severe mental disorder from the time when it was first diagnosed. Finally, it would had been interesting to measure what produced the variations in SQOL between T0 and T1 or to identify predictors of SQOL decrease or increase in T1, which is another relevant question. Other studies are needed to complement our research with regard to these aspects.

## Conclusion

This longitudinal study is one of the first to test such a large number of variables that can predict SQOL in a cohort of users with severe mental disorders. Based on previous research, a comprehensive conceptual framework was developed that summarized possible predictors of SQOL. Further studies could build on this framework. This study is also among the few to compare SQOL within a heterogeneous cohort of users representing the full spectrum of serious mental disorders and living in the community. It found that the severity of needs is the strongest SQOL predictor among this group and that SQOL is most often associated with health needs. Meeting these serious needs should be the priority of mental health services. Moreover, a moderate problem in the social needs category is enough to predict a negative SQOL – which is a key finding as most studies have essentially focused on the importance of unmet needs (or serious problems). Specifically, company was the area that most significantly altered SQOL. Furthermore, when compared with users affected by other severe mental disorders, users with schizophrenia had a better SQOL. Users living in supervised housing had better SQOL than those living in autonomous housing. Finally, the study confirms the significant impact on SQOL of social integration and reassurance of worth – two aspects of social support. Priority should be given to programs and interventions that promote reliable and supportive social networks enabling social integration, reassurance of worth, and feelings of personal safety. In addition, residential services matching the level of functioning of users should be maintained or developed.

## Abbreviations

ACSS-MH: Alberta Continuity of Services Scale for Mental Health; AUDIT: Alcohol use disorders identification test; CAN: Camberwell assessment of need; CCHS: Canadian community health survey; DAST-20: Drug abuse screening test-20; HSSC: Health and social services centers; MANQ: Montreal assessment of needs questionnaire; MCAS: Multnomah community ability scale; MHUI: Mental Health University Institute; QOL: Quality of life; SUQ: Service utilization questionnaire; SLDS: Satisfaction with life domains scale; SPS: Social provision scale; SQOL: Subjective quality of life.

## Competing interests

The authors declare that they have no competing interests.

## Authors’ contributions

MJF and GG designed the study. JMB carried out the statistical analyses with assistance from JC, NS and JT. MJF and GG wrote the article. All authors have read and approved the final manuscript.
